# Dr. Reynell Coates’ Contributions to Surgery: Note on the Means of Avoiding Excoriations of the Heel and Perineum in Fractures of the Lower Extremities, Treated by Permanent Extension

**Published:** 1842-04-23

**Authors:** Reynell Coates


### *Contributions to Surgery.*

BY REYNELL COATES, M. D.

*Note on the means of avoiding Excoriations of the Heel and Perineum in Fractures of the Lower Extremities, treated by Permanent Extension.*—The experience of the Pennsylvania Hospital during the House-surgeoncy of Drs. J. R. Barton, B. H. Coates, Ritchie and the writer, covering a period of more than ten years, sufficiently established the fact that, with due care and a close attention to little niceties in dressing, fractures of the thigh may be generally treated in the extended position without danger of excoriation. Yet, with certain forms of apparatus, abrasion, and sometimes deep ulceration to an exceedingly embarrassing extent occurred in a large majority of cases. From the time of John Bell's philippics against Dessault and Physick—the great advocates of the extended position—down to the present moment, the accidents just mentioned have furnished the most forcible arguments of the enemies of the method ; and my attention was drawn, very early in professional life, to the investigation of the true causes of these excoriations. Firmly believing them all to be entirely independent of the essential actions of the apparatus, and due exclusively to errors of construction in the minor details, or to seemingly slight, but really important faults of manipulation, I have no hesitation in expressing the opinion that ulceration or excoriation may be not only avoided by proper precautions in almost every case, but that, in most instances, when they have been already produced by a want of care, they may be healed without much difficulty, without any important intermission of the extension of the limb. Let us then examine the causes of these dreaded accidents.

In the eleventh number of this volume, (p. 164,) I have spoken of the slightness of the effort required to effect the gradual but rapid reduction of fractures of the thigh, and the great increase of the force necessary when the muscles of a shortened limb have been allowed to accustom themselves to their contracted condition for several hours. If, then, we employ extending or counter-extending bands of yielding materials, we must calculate upon their becoming stretched from time to time so as to permit a certain degree of overlapping of the fragments from muscular contraction, and an inevitable increase of the force required for ultimate reduction. The enhanced pressure thus rendered necessary is among the principal causes of excoriation.

The articles usually employed for the purpose of extension, in the Pennsylvania Hospital, have been the simple or stuffed buckskin gaiter with its well known appendages of tape, the folded silk handkerchief, and the peculiar bands introduced by myself in 1820. The gaiter, in its simplest form, is a piece of soft buckskin made to cover the tendo Achillis, and—sweeping around the ankle so as to cover the malleoli,—to meet on the front of the leg and top of the instep, where it is secured by lacing with a thong of the same material, passed through a series of eyelet holes, and intercrossed over a tongue. The lateral parts of this gaiter sweep downward by a convex curve, until their edges reach a little below the sole. To a worked eyelet hole on each side is appended a long piece of tape, to be employed as the direct means of extension ; and the tapes are used, for this purpose, with or without the aid of a foot-board, screw, spring or weight, according to the peculiar mechanical notions of the attending surgeons. But these last named pieces of apparatus will be reviewed hereafter.

As the lateral eyelet holes are the only portions of the gaiter which require preparation by the needle, no stitch or other irritating projection is presented to the skin ; and nothing can be more bland in its action than a dressing of soft buckskin applied directly to the cuticle. Moreover, it becomes very readily stretched, so as to adapt itself pretty closely to the form of the parts which it covers. But there are two serious inconveniences attendant on the use of this most popular of all extending bands. Firstly ; it prevents the accurate admeasurement of the limb, which cannot be effected otherwise than by ascertaining the distance between the superior spinous process of the ilium and the point of one of the malleoli ; and, secondly ; the material yields so readily to extension, that it is incapable of resisting the contractile force of the great muscles of the thigh in a vigorous patient. It is true that this disposition to stretch under the action of the tapes continually decreases from day to day ; but it is so considerable for several days that the surgeon is compelled to pay almost hourly attention to the condition of the bands during the first week, if he wish to prevent considerable shortening, to which the muscles are continually becoming accustomed during his absence. For this reason, also, there is a perpetual temptation to employ undue extending force to compensate for the yielding of the gaiter. Again: as the leather loses its capacity for stretching, it is exceedingly apt to be thrown into irregular folds on the back of the heel and summit of the instep, where the pressure is greatest ; and these folds are exceedingly fertile sources of excoriation.

The value of the simple buckskin gaiter may be briefly stated thus :—In hospital practice, when great caution is used by a resident surgeon, a fractured thigh may be brought to the full length by its means in a majority of cases, and excoriations of the heel do not very often occur ; but the management of the case is generally troublesome to the operator, and very annoying to the patient, from the frequent occasion for examination and measurement ;—the latter always requiring an intermission of the extending force, if not the unlacing of the gaiter. In private practice, though several times called to see cases in which the limb was stated to be of the full length after the union was far advanced, I have not yet failed to detect, in every case treated by the simple gaiter, nearly half an inch of shortening. The proportion of cases of excoriation—though rarely to serious extent—has been very large in private life, and no inconsiderable share of the evil appears to me fairly chargeable to the intrinsic properties of this part of the apparatus.

(*To be continued.*)

